# Thoracoscopic Resection of a Bronchial Artery Aneurysm Mimicking Mediastinal Lymph Node Metastasis after Surgery for Papillary Thyroid Cancer: A Case Report

**DOI:** 10.70352/scrj.cr.26-0069

**Published:** 2026-04-01

**Authors:** Yoshihiro Shimomura, Norihisa Uemura, Daisuke Hayashi, Koki Tabata, Tetsuo Tsukahara, Takaaki Ito, Takeshi Amemiya, Satomi Saeki, Toshiyuki Arai

**Affiliations:** Anjo Kosei Hospital, Anjo, Aichi, Japan

**Keywords:** bronchial artery aneurysm, aneurysmectomy, thoracoscopic surgery

## Abstract

**INTRODUCTION:**

Mediastinal lymph node recurrence is frequently encountered during the follow-up after surgery for thyroid cancer. We report a rare case of a bronchial artery aneurysm (BAA) that was preoperatively suspected to be a mediastinal lymph node metastasis based on imaging findings and subsequently treated with thoracoscopic resection.

**CASE PRESENTATION:**

A 68-year-old male underwent a total thyroidectomy and neck lymph node dissection for papillary thyroid carcinoma (pT1bN1bM0, UICC Stage II), followed by radioactive iodine ablation as adjuvant therapy. Surveillance CT performed 2 years postoperatively revealed a 30-mm mass in the subcarinal region of the mediastinum. The mass showed peripheral calcification with an internal low-density area. Serum thyroglobulin level was 0.18 ng/mL (within normal limits). PET revealed mild fluorodeoxyglucose uptake with a maximum standardized uptake value of 2.5. These findings were consistent with solitary mediastinal lymph node metastasis. No other findings suggestive of recurrence were identified, leading to the decision to perform thoracoscopic resection of the mediastinal tumor for both diagnostic confirmation and therapeutic intervention. The operative time was 67 min with minimal blood loss, and the patient was discharged on POD 3 without complications. Histopathological examination revealed that the mass was a 25-mm arterial aneurysm with atherosclerotic changes and no evidence of malignancy.

**CONCLUSIONS:**

When evaluating a subcarinal mediastinal mass following thyroid cancer surgery, it is important to include vascular lesions, such as BAAs, in the differential diagnosis, although these are rare. This case demonstrates that minimally invasive thoracoscopic resection provides both diagnostic confirmation and therapeutic benefits when preoperative diagnosis remains uncertain.

## Abbreviations

ATA
: American Thyroid Association
BAA
: bronchial artery aneurysm
EBUS-TBNA
: endobronchial US-guided transbronchial needle aspiration
FDG
: fluorodeoxyglucose
PTC
: papillary thyroid carcinoma
RAI
: radioactive iodine
SUVmax
: maximum standardized uptake value
TAE
: transcatheter arterial embolization
WBS
: whole-body scan

## INTRODUCTION

Mediastinal lymph node recurrence is frequently encountered during surveillance of patients following surgical treatment for thyroid cancer. The incidence of mediastinal lymph node metastases in PTC ranges from 0.7% to 27%.^1–8)^ During postoperative surveillance, CT and PET are commonly used to detect recurrent disease. However, distinguishing malignant lymph node metastases from benign mediastinal masses remains a significant diagnostic challenge. BAA is a rare disease, with a reported incidence of < 1% on selective bronchial angiography.^9)^ BAA is classified as intrapulmonary, mediastinal, or multiple, based on its anatomical location. Mediastinal BAA is even rarer, with only a few cases reported in the literature. The clinical presentations of BAA vary widely, ranging from incidental radiological findings to catastrophic rupture with hemorrhagic shock.^10–12)^ Early treatment is essential once it is diagnosed. We present the case of a 68-year-old man with a mediastinal BAA that was initially suspected to have lymph node recurrence following surgical treatment for PTC. The aneurysm was incidentally detected and was successfully managed via thoracoscopic resection.

## CASE PRESENTATION

**Fig. 1:**
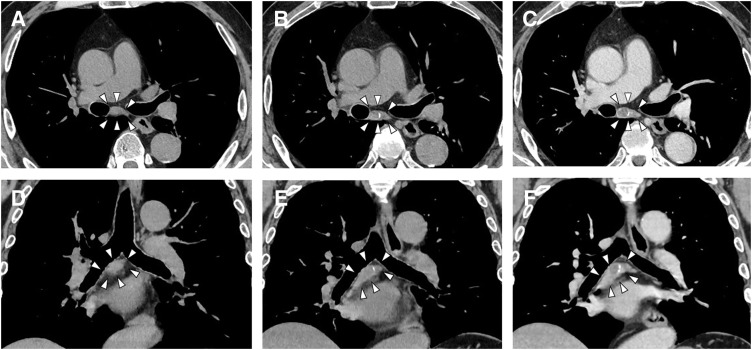
A mass (arrowheads) in the subcarinal region of the mediastinum. (**A**, **D**, non-contrast) CT images obtained at the time of surgery for thyroid cancer. (**B**, **C**, non-contrast) and (**E**, **F**, contrast-enhanced) CT images obtained 2 years after surgery.

**Fig. 2:**
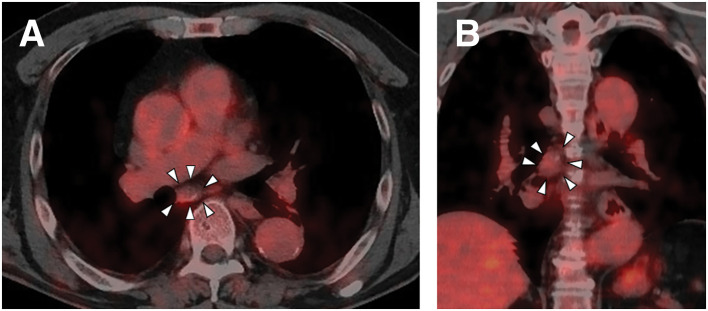
PET showing the mild FDG uptake (SUVmax 2.5) of the mass (arrowheads). (**A**) Axial PET image. (**B**) Coronal PET image. FDG, fluorodeoxyglucose; SUVmax, maximum standardized uptake value

**Fig. 3:**
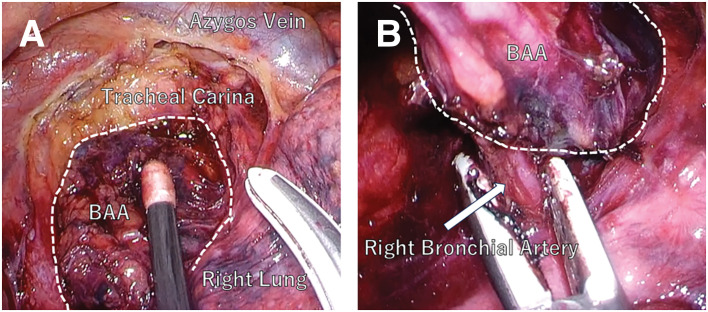
Thoracoscopic findings. A subcarinal mass (dotted lines) with an identifiable right bronchial artery (arrow). (**A**) Before dissection. (**B**) After dissection. BAA, bronchial artery aneurysm

**Fig. 4:**
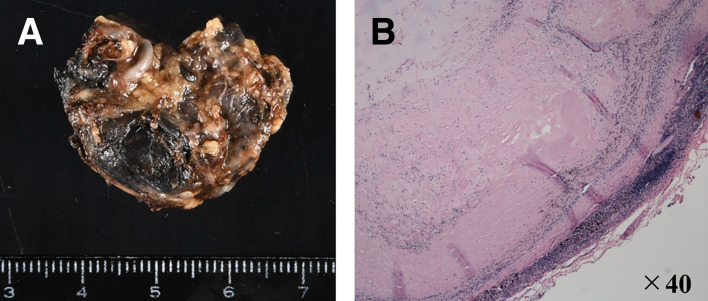
(**A**) Macroscopic findings. The size was 30 × 25 mm. (**B**) Hematoxylin and eosin staining (original magnification ×40). An arterial structure with atherosclerotic plaque.

A 68-year-old male underwent a total thyroidectomy with bilateral neck lymph node dissection for PTC (pT1bN1bM0, UICC Stage II). Following surgery, the patient underwent RAI ablation as adjuvant therapy. During postoperative surveillance, the patient remained asymptomatic with thyroglobulin suppression. Surveillance CT performed 2 years postoperatively detected a 30-mm mass in the subcarinal region of the mediastinum. The mass showed slight enlargement with development of peripheral calcification and internal low-attenuation changes (**Fig. 1**). Serum thyroglobulin level was 0.18 ng/mL (within normal limits). PET showed mild FDG uptake (SUVmax 2.5) (**Fig. 2**), which was insufficient to definitively exclude malignancy. Because mediastinal lymph node metastasis could not be excluded based on the clinical course and imaging findings, thoracoscopic resection was performed for both definitive diagnosis and treatment. A 4-port technique (two 12-mm ports and two 5-mm ports) was used via the right thoracic approach with the patient in the prone position under single-lung ventilation. A 30-mm mass detected in the subcarinal region (**Fig. 3A**) was carefully dissected from the mediastinal structures. A relatively large feeding artery (**Fig. 3B**) was identified and securely controlled using an ultrasonic coagulation device. The mass was excised en bloc (**Fig. 4A**). A summary video of the surgical procedure is provided as **Supplementary File 1**. Macroscopically, the mass presented as an enlarged blackened lymph node, consistent with metastasis. Had an aneurysm been suspected, clip ligation of the artery might have been the safer and more reliable method of vascular control. The operation was performed under the supervision of a board-certified esophageal surgeon. The operative time was 67 min, with minimal blood loss. Intraoperative complications were not observed. The patient was discharged on POD 3 without complications. A histopathological examination revealed a 25-mm arterial aneurysm with atherosclerotic changes (**Fig. 4B**). The diagnosis was confirmed as idiopathic mediastinal BAA with atherosclerotic degeneration.

## DISCUSSION

In high-risk patients, according to the ATA risk categories, who underwent thyroidectomy for PTC, the recurrence rate has been reported as 22.7%.^13)^ The incidence of mediastinal lymph node metastases in PTC ranges from 0.7% to 27%.^1–8)^ The imaging characteristics observed in the patient (a 30-mm mass with peripheral calcification, central low-attenuation areas, and mild FDG uptake [SUVmax, 2.5]) were consistent with lymph node metastasis. Mediastinal masses on surveillance imaging in patients with a history of thyroid cancer naturally raise the suspicion of lymph node metastasis. However, various diseases may exhibit similar imaging findings. For example, calcification of mediastinal lymph nodes can occur in diseases such as lymphoma, metastatic carcinoma, tuberculosis, and sarcoidosis.^14)^ It is essential to distinguish benign from malignant masses. Although chronological imaging changes play an important role in diagnostic assessment, contrast-enhanced CT and PET were not performed at the time of the initial surgery. Consequently, comparison of imaging findings between the initial and current examinations was insufficient, which limited the evaluation of temporal changes. PET is typically used to differentiate benign and malignant diseases. Unfortunately, FDG is not specific to the detection of malignant conditions. Inflammatory cells also show avidity on FDG.^15)^ Yu et al. reported that SUVmax was not helpful in differentiating between benign and malignant lesions in patients with enlarged mediastinal lymph nodes.^16)^ The 2025 ATA Management Guidelines for Adult Patients with Differentiated Thyroid Cancer indicate that the frequency of false-positive lesions on PET varies from 0% to 39%. These false-positive rates justify a fine-needle aspiration biopsy with cytology and thyroglobulin measurement in the hub washout for cases where an accessible lymph node is identified.^17)^ In this case, extensive lymph node metastasis was observed at the initial surgery, leading us to primarily suspect mediastinal lymph node recurrence and proceed with surgical resection. Given that the lesion was located in the subcarinal region, EBUS-TBNA might have represented a feasible initial diagnostic option. If EBUS-TBNA had demonstrated no evidence of malignancy, short-term radiological imaging surveillance could also have been considered as an alternative management strategy. The sensitivity of EBUS-TBNA for the evaluation of mediastinal lymph nodes has been reported to be 81%–97% in non–small cell lung cancer and 84% in sarcoidosis.^18,19)^ Although the risk of false-negative results with EBUS-TBNA is relatively low, such results may lead to delayed treatment and thus should not be overlooked. The complication rate of EBUS-TBNA has been reported to be 1.23%, with hemorrhage being the most common complication, occurring in 0.68% of cases.^20)^ Retrospectively, the mass proved to be an aneurysm, and biopsy could have entailed a potential risk of bleeding, warranting particular caution. EBUS-TBNA should be considered as a safe and reliable minimally invasive diagnostic option for subcarinal mediastinal mass when metastasis is suspected. Other imaging modalities available include RAI WBS. WBS may be considered in patients at intermediate-high to high risk of recurrence when there is clinical suspicion of recurrence, to evaluate for iodine-avid disease.^17)^ However, following RAI ablation or adjuvant therapy, when the post-therapy WBS does not reveal uptake outside the thyroid bed, subsequent diagnostic WBS has low sensitivity.^17)^ In this case, given that post-ablation WBS showed no radioiodine uptake outside the thyroid bed, diagnostic WBS was deemed to have low utility and was therefore not performed. BAA is a rare vascular lesion that is often asymptomatic and detected incidentally during imaging surveillance for other conditions. The etiology of BAA remains multifactorial and often obscure. Reported predisposing factors include atherosclerosis, chronic pulmonary inflammation, trauma, pulmonary agenesis, bronchiectasis, and vascular abnormalities, such as Osler–Weber–Rendu and Behcet diseases.^21,22)^ In the patient, the aneurysm was associated with atherosclerotic changes, suggesting that arteriosclerosis contributed to aneurysm formation. BAA is often detected on CT, and a definitive diagnosis is established using selective bronchial artery angiography. In our case, due to thrombotic occlusion, the aneurysmal lumen showed no contrast enhancement, and its location led to the suspicion of a lymph node rather than an aneurysm. There have been reported cases where BAAs were initially diagnosed as mediastinal malignancy or benign esophageal tumor.^9,23)^ As a characteristic CT finding in BAAs, the presence of an internal low-attenuation rounded structure adjacent to the vessels has been reported.^9)^ The attenuation varies depending on chronicity and changes over time.^24)^ Once diagnosed, mediastinal BAAs require treatment, as rupture cannot be predicted based on aneurysm size.^22)^ Treatment options include TAE and surgical resection (open thoracotomy or thoracoscopy). TAE has recently become the first-line treatment for BAA because it is minimally invasive and can be performed without general anesthesia.^11,25)^ However, TAE can be challenging or even infeasible in certain cases, such as when the proximal segment of the feeding artery is too short for a stable embolization. Surgical resection permits the complete removal of the aneurysm and a definitive histological diagnosis. Historically, open thoracotomy was the mainstay of treatment, but thoracoscopic surgery has been reported.^26–29)^ More recently, resection using robotic surgery has also been described.^30)^ While conventional open thoracotomy may be favored in life-threatening hemorrhage, thoracoscopic resection should be considered a minimally invasive treatment for asymptomatic patients for whom catheter embolization is challenging. This case emphasizes critical points for managing patients with postoperative mediastinal masses following thyroid cancer surgery. First, the differential diagnosis must remain broad; not all mediastinal masses are lymph node metastases, and vascular lesions, although rare, should be included in the diagnostic algorithm. Second, detailed vascular imaging, including angiography, should be considered when imaging features are atypical or when a lesion exhibits anatomical features suggestive of vascular origin.

## CONCLUSIONS

When evaluating subcarinal mediastinal masses, clinicians must consider rare vascular lesions, such as BAA, in the differential diagnosis. Given the risk of catastrophic rupture and the high mortality associated with BAA, an early diagnosis and intervention are essential. Bronchial artery angiography is valuable in suspected vascular pathologies. This case demonstrates that when the preoperative diagnosis remains uncertain, minimally invasive thoracoscopic resection provides both diagnostic confirmation and therapeutic benefits.

## SUPPLEMENTARY MATERIAL

Supplementary File 1 Summary video of the surgery. Thoracoscopic video of the subcarinal mediastinal area in the prone position.

## DECLARATIONS

### Availability of data and materials

The datasets supporting the conclusions of this article are included within the article.
